# Systematic review and meta-analysis of laparoscopic mesh versus suture repair of hiatus hernia: objective and subjective outcomes

**DOI:** 10.1007/s00464-017-5586-x

**Published:** 2017-05-18

**Authors:** Chao Zhang, Diangang Liu, Fei Li, David I. Watson, Xiang Gao, Jan H. Koetje, Tao Luo, Chao Yan, Xing Du, Zhonggao Wang

**Affiliations:** 1 Department of General Surgery, Xuanwu Hospital, Capital Medical University, No. 45 Changchun Street, Xicheng District, Beijing, 100053 China; 2 Flinders University Department of Surgery, Flinders Medical Centre, Bedford Park, SA 5042 Australia

**Keywords:** Hiatus hernia, Hiatal repair, Mesh, Gastroesophageal reflux disease (GERD), Quality of life (QOL)

## Abstract

**Background:**

Hiatus hernia (HH) contributes to the pathophysiology of gastroesophageal reflux disease (GERD). Mesh-augmentation of surgical repair might be associated with a reduced risk of recurrence and GERD. However, recurrence rates, mesh-associated complications and quality of life (QOL) after mesh versus suture repair are debated. The aim of this meta-analysis was to determine HH recurrence following mesh-augmentation versus suture repair. Secondary aims were to compare complications, mortality, QOL and GERD symptoms following different repair techniques.

**Methods:**

A systematic literature search of the PubMed, Medline, Embase, Cochrane Library, and Springer database was performed to identify relevant studies comparing mesh-augmentation versus suture repair of the esophageal hiatus. Data pertinent to the benefit versus risk outcomes for these techniques were extracted and compared by meta-analysis. The odd ratio (OR) and mean differences (MD) with 95% confidence intervals were calculated.

**Results:**

Eleven studies (4 randomized, 9 non-randomized) comparing mesh (*n* = 719) versus suture (*n* = 755) repair were identified. Mesh-augmentation was associated with a reduced overall recurrence rate compared to suture repair [2.6 vs. 9.4%, OR 0.23 (95% CI 0.14–0.39), *P* < 0.00001]. There was no significant difference in the incidence of complications (*P* = 0.400) between groups. Improvement in QOL measured by SF-36 was greater following biological mesh-augmentation compared to suture repair (MD = 13.68, 95% CI 2.51–24.85, *P* = 0.020), as well as GERD-HRQL. No differences were seen for the GIQLI scores with permanent mesh (*P* = 0.530). Dysphagia improvements were better following suture repair (MD = 1.47, 95% CI 0.20–2.74, *P* = 0.020).

**Conclusions:**

Mesh repair of HH conferred some advantages and disadvantages at short-term follow-up. Compared to a suture repair alone, mesh-augmentation might be associated with less short-term recurrences, and biological mesh was associated with improved short-term QOL. However, these advantages were offset by more dysphagia. Long-term outcomes are still needed to determine the place of mesh repair of HH.

While laparoscopic fundoplication is a well-established option for the treatment of gastroesophageal reflux disease (GERD), controversy exists about the best technique for repair of hiatus hernia (HH), and how to minimize the risk of hernia recurrence [1]. Mesh-augmented hiatal repair has been proposed as a solution which might reduce the risk of recurrence, and a range of different techniques, mesh shapes, and mesh types has been proposed [2, 3]. The main indication for mesh-augmented hiatal repair is a large paraesophageal hernia. However, some surgeons also advocate the routine use of mesh repair for smaller sliding HHs [4]. On the other hand, the disadvantages of mesh-augmentation include the risk of serious complications related to the use of prosthetic material, such as esophageal erosion and stenosis, mesh migration, local fibrosis, and dysphagia [5]. Even with the use of biological mesh, postoperative dysphagia is still reported in 12–17.5% of patients [6]. For these and other reasons, mesh-augmentation of HH repair remains controversial.

If mesh repair is to be routinely adopted into surgical practice, its use should minimize the risk of hernia recurrence without increasing the risk of complications and other adverse outcomes. It is also possible that subsets of patients might benefit from mesh repair whereas others might not. For example, the size of the hernia or hiatal defect might be an important factor in determining the need or otherwise for mesh [7, 8]. Previous literature reviews [7, 9] have summarized available data for hernia recurrence following primary suture repair versus mesh-augmentation using various mesh types, but without analyzing the impact of hernia size on outcome. In addition, while current evidence [10, 11] suggests mesh-augmentation is not associated with an increase in risk of perioperative complications, previous systematic reviews [11] have not considered the issue of postoperative dysphagia or other issues which can occur following both suture repair and mesh-augmentation. These previous meta-analyses [9, 11] have all focused on the scope of objective investigations and hernia recurrence rates, and subjective outcomes such as GERD symptoms and global outcome measures have not been considered.

In this paper, we report the outcomes for a systematic review of the literature and meta-analysis of outcomes following laparoscopic mesh-augmentation versus suture repair of HH. Clinical outcomes were compared following surgical repair, including recurrence rates, complications, and post-repair dysphagia. We also reviewed the impact of hernia size on outcome to determine whether recurrence is impacted by hernia size following hiatal repair with mesh-augmentation. Finally, the impact of different methods of repair on quality of life (QOL) outcomes was also determined.

## Materials and methods

This meta-analysis was conducted and the results are presented according to the recommendations of PRISMA statement [12].

### Search strategy

PubMed, Medline, Embase, Cochrane Library, and Springerlink electronic databases were searched until October 2016. A manual search of the reference listed from the articles accessed was also performed to identify other relevant studies. Only articles written in English were searched. A search strategy using disease-specific terms (e.g., gastroesophageal reflux OR hernia, hiatal OR hiatus hernia), management-specific terms (e.g., mesh OR implant OR patch), and terms related to surgical procedures (e.g., laparoscopic surgery OR laparoscopy OR minimally invasive) was used.

### Inclusion criteria and exclusion criteria

Inclusion criteria were as follows: (i) randomized controlled trials (RCTs) or case–control comparative studies in patients who underwent repair of HH with the use of mesh (permanent or absorbable/biologic), (ii) age ≥16 years, (iii) a laparoscopic approach was used in all patients, (iv) duration of follow-up ≥12 months, (v) raw data could be extracted from studies to calculate outcomes, (vi) patients were diagnosed preoperatively with GERD or any type of HH. Exclusion criteria were as follows: (i) Studies were non-comparative, (ii) mesh was not used, (iii) the hernia was repaired without a fundoplication, (iv) patients aged <16 years were included, (v) studies for which raw data could not be extracted to obtain pooled results and the corresponding author was not able or willing to provide revelent additional data at request for this study.

### Data extraction

Titles and abstracts of the citations were identified and scrutinized by two of the authors (C Zhang and D Liu) to determine eligibility for inclusion in the pooled analysis. Data were then extracted from the full publication. This comprised (i) descriptive information relevant to each study—first author, publication year, study population characteristics, study design, sample size, follow-up duration, and inclusion/exclusion criteria, and (ii) data required for outcomes analysis, including beneficial and adverse results. Any disagreements between the two reviewers were resolved by discussion and consensus. If data were missing, the authors of the original studies were contacted and the relevant information was requested. Outcomes of interest in studies in which the same cohort was published in more than one paper were extracted based on the article that was published most recently.

### Outcomes of interest and evidence synthesis

Two study groups were defined and compared: (1) patients who underwent a primary suture repair of esophageal hiatus without mesh, and (2) patients who had hiatal repair with a mesh-augmentation technique. Five outcome parameters were synthesized and described for this meta-analysis:

1. Recurrence of HH, with subgroup analysis for different HH sizes.
2. Total complications, and the incidence of dysphagia.
3. Review of mortality and its cause.
4. Visual analog scales (VAS) assessing postoperative symptoms, including heartburn, regurgitation, non-cardiac chest pain and dysphagia.
5. Meta-analysis of QOL measured by the Short-Form 36 (SF-36), Gastrointestinal Quality of Life Index (GQOLI) and GERD related Health Related Quality of Life (HRQL) questionnaire.

### Methods of analysis

Data extracted from eligible trials were integrated using Review Manager 5.3 (The Nordic Cochrane Centre, Copenhagen, Denmark) provided by the Cochrane Collaboration. Additional statistical expertise was provided by an author—X Du. Outcomes reported in two or more studies were pooled for meta-analysis. Dichotomous and continuous outcomes were presented as odds ratio (OR) and mean differences (MD) respectively. Pooled OR with 95% confidence intervals (CI) were calculated using the Mantel–Haenszel model to measure the effect of each type of repair on the risk for hernia recurrence and complications, and continuous variables were pooled using the inverse variance method to compare symptom scales and QOL. Heterogeneity was assessed using the *I* ^2^ statistic, a method expressing the percentage of variation across studies. *I* ^2^ values between 0 and 25% were considered to suggest a low level of variation, values above 25% a moderate level, and values above 75% a high level of heterogeneity. The fixed-effects model was used if heterogeneity was absent (*χ* ^2^ test, *P* > 0.1, and *I* ^2^ < 50%), otherwise the random-effects model was used [13]. In addition, the random-effects model was applied to synthesize summative data to compensate for heterogeneity of non-randomized studies. Subgroup analysis was performed to assess the recurrence rate of HH and QOL.

## Results

### Description of studies

**Fig. 1:**
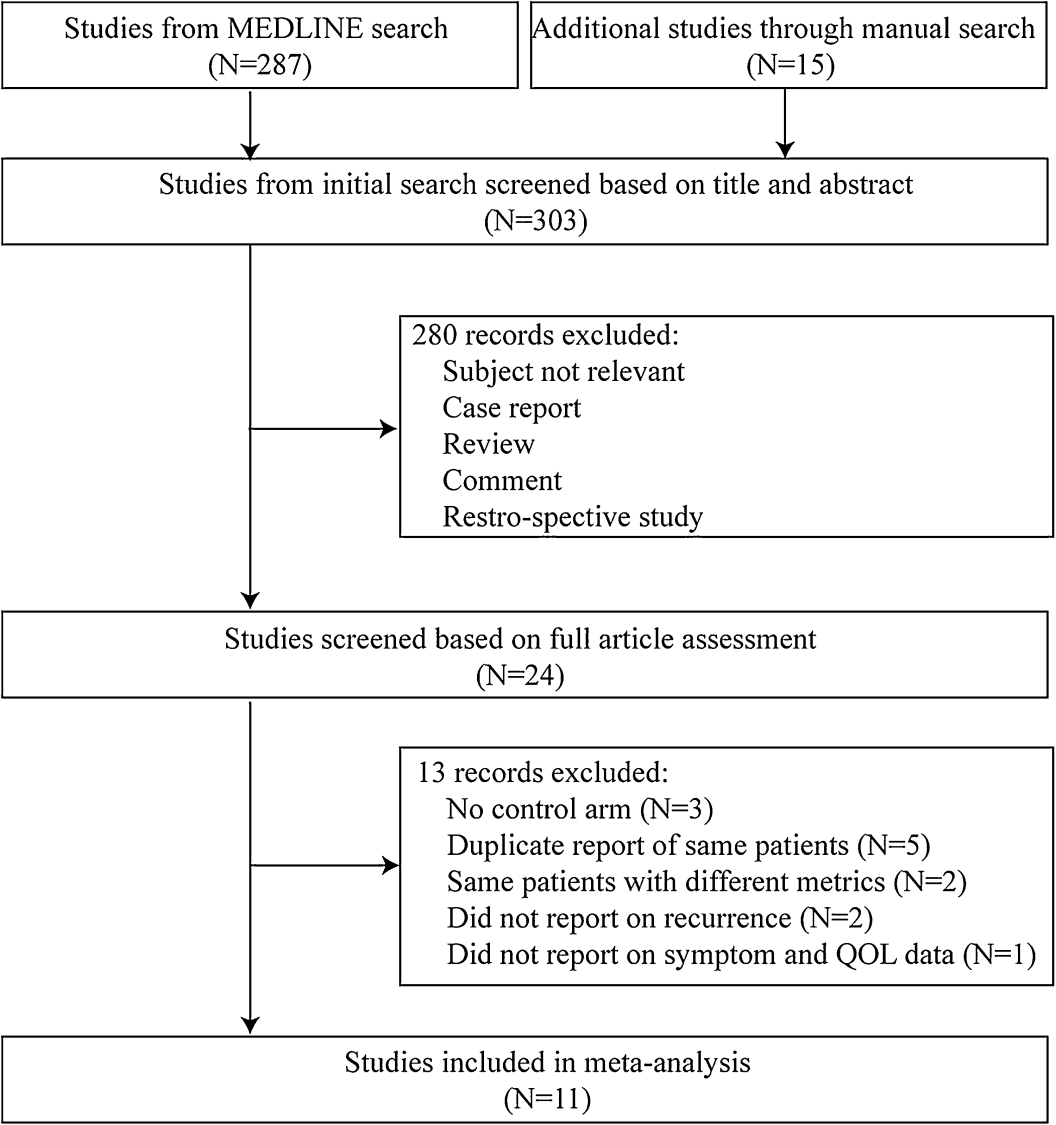
Flow chart summarizing literature assessment

Figure 1 shows the PRISMA flow chart for the literature search. The initial database search retrieved 303 publications, and 11 eligible publications met the inclusion criteria, including four RCTs [14–17] three retrospective case–control [18–20] and four prospective case–control studies [21–24]. In two of the RCTs, different metrics from the same patients were described, and the data from these trials were included once in the meta-analysis. In the other two trials, different follow-up times were reported and both were included in the meta-analysis [16, 17, 25, 26]. Basic characteristics of the included studies are summarized in Table 1, including case number, patient age, male–female ratio, follow-up time, and inclusion criteria. In total 1474 patients were included, 719 with mesh-augmentation and 755 with primary suture repair. Included studies were published between 2002 and 2016. Duration of follow-up ranged from 6 to 58 months. Not all studies provided data relevant to the outcomes of interest.

**Table 1:** The basic characteristics of included randomized clinical trials

Author	Years of publication	Study type	Country	*N* (mesh/suture)	Sex ratio (male/female)	Age (total or mesh/suture)	Detail of mesh material	Follow-up (months)
Asti [21]	2016	Prospective trial	Italy	41/43	20/64	66/66	Biological mesh	24
Crespin [18]	2016	Retrospective trial	United States	110/36	34/112	61.5	Biological mesh	9
Frantzides [14]	2002	RCT	United States	36/36	NA	63/58	Permanent mesh	30
Granderath [15]	2005	RCT	Germany	50/50	62/38	48/49	Permanent mesh	12
Kamolz [19]	2002	Retrospective trial	Austria	100/100	121/79	48/50	Permanent mesh	12
Oeschager [16, 25]	2006, 2011	RCT	United States	51/57	27/81	67/64	Biological mesh	6/58
Ozmen [22]	2014	Prospective trial	Turkey	31/29	34/26	41/42	Permanent mesh	12
Ringley [23]	2006	Prospective trial	United States	22/22	24/20	58/52	Biological mesh	6
Schmidt [20]	2014	Retrospective trial	United States	38/32	29/41	51/41	Biological mesh	12
Kepenekci [24]	2007	Prospective trial	Turkey	176/335	271/240	48	Permanent mesh	24
Watson [17] and Koetje [26]	2015	RCT	Australia	83/43	40/86	68/68	Biological/Permanent mesh	12

*RCT* randomized controlled trials

### Risk of bias within studies

The study design (RCTs, prospective, or retrospective) and study characteristics are summarized in Table 1. Potential sources of bias, other than design, are summarized in Table 2. The main limitations resulted from poor description of the randomization processes [14, 15, 22] as well as a lack of (or poor description of) the blinding processes [14, 15].

**Table 2:** Risk of bias summary

	①	②	③	④	⑤	⑥	⑦
Asti 2016	LR	UR	UR	LR	LR	UR	LR
Crespin 2016	UR	UR	UR	LR	LR	LR	LR
Frantzides 2002	UR	UR	UR	LR	LR	LR	LR
Granderath 2005	UR	UR	UR	LR	LR	LR	LR
Kamolz 2002	UR	UR	UR	LR	LR	LR	LR
Oeschager 2006, 2011	LR	LR	LR	LR	LR	LR	UR
Ozmen 2014	LR	UR	UR	LR	LR	LR	LR
Ringley 2006	UR	UR	UR	LR	LR	LR	LR
Schmidt 2014	UR	UR	UR	UR	LR	LR	LR
Kepenekci 2007	UR	UR	UR	LR	LR	UR	LR
Watson and Koetje 2015	LR	LR	LR	LR	LR	LR	LR

①:Random sequence generation; ②:Allocation concealment; ③:Blinding of participants and personnel ④:Blinding of outcomes assessment; ⑤:Incomplete outcome data; ⑥:Selective reporting; ⑦:Other bias. *LR* low risk, *UR* unclear risk, *HR* high risk

### Recurrence of HH

**Fig. 2:**
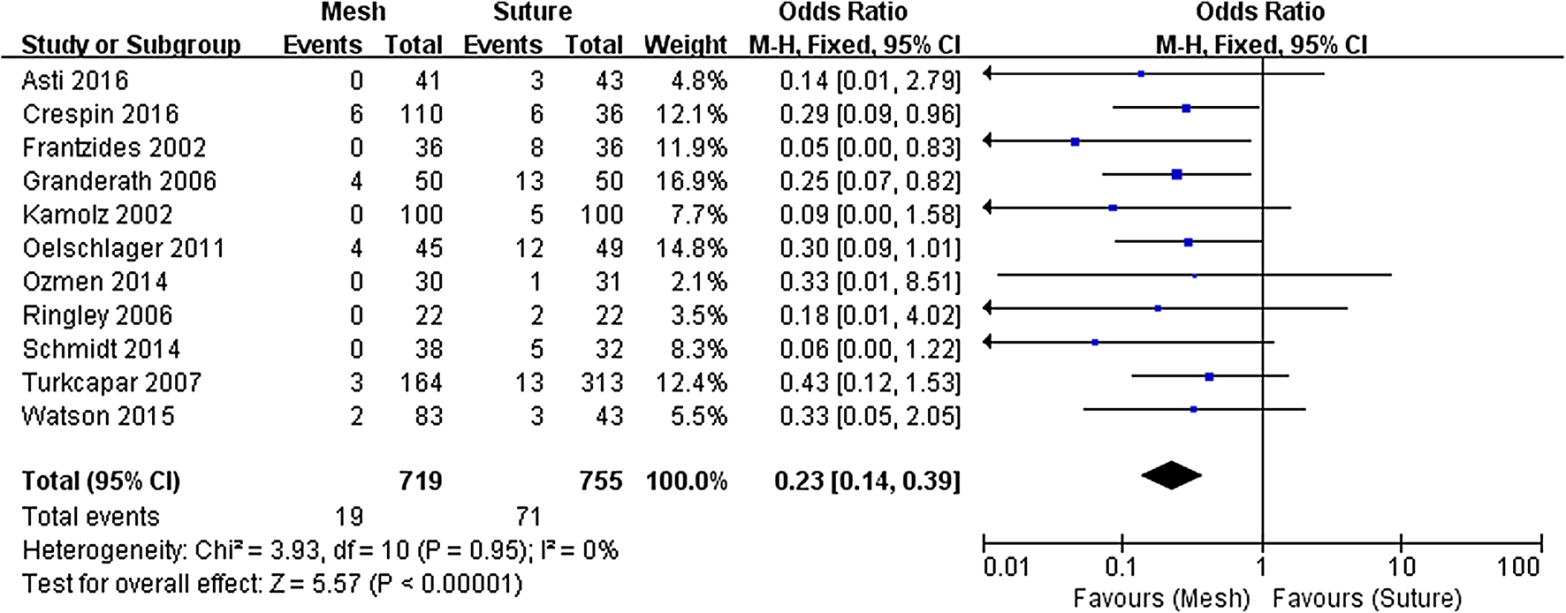
Forrest plot of the odds ratio for early recurrence

Clinical recurrence at short- to middle-term follow-up (6–36 months), defined as symptomatic recurrence, was evident in 2.6% of patients following mesh-augmented repair versus 9.4% following a suture repair (OR 0.23, 95% CI 0.14–0.39, *P* < 0.00001), with no evidence of statistical heterogeneity (*χ* ^2^ = 3.93, *P* = 0.950, *I* ^2^ = 0%). The Forest plot of odds ratios for the risk of hernia recurrence is presented in Fig. 2. Long-term (5 year) recurrence data were only available from one of the RCTs [25] and no significant differences were seen for mesh (14/33, 42.4%) versus suture repair (20/39, 51.3%, OR 0.70, 95% CI 0.28–1.78, *P* = 0.450).

**Fig. 3:**
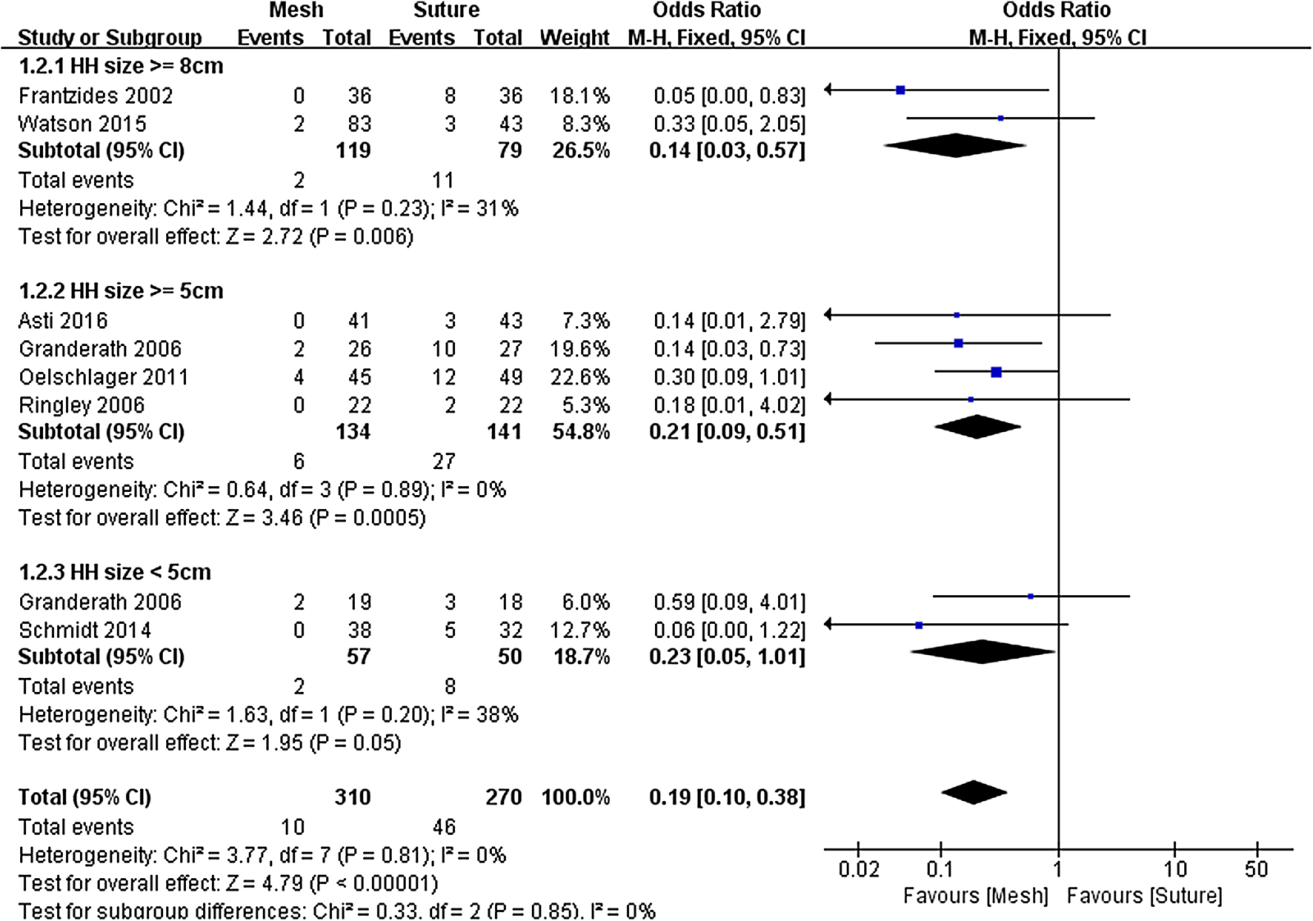
Forrest plot of the subgroup odds ratio for early recurrence by HH size

To evaluate the impact of HH size on hernia recurrence rates, subgroup analyses were conducted. For these analyses, HHs were grouped into 3 size categories based on endoscopically measurements: very large (more than 8 cm), large to very large (more than 5 cm), and small (less than 5 cm). Two studies with very large HH, four studies with large to very large HH and two studies with small HH were included in subgroup meta-analysis. When total-group analyses were performed, the meta-analysis revealed a significant difference between the two arms for this parameter (OR 0.19, 95% CI 0.10–0.38, *P* < 0.00001, *I* ^2^ = 0%). In the subgroup with small, large to very large, and very large HH, the results also favored mesh-augmented repair (Fig. 3) (Very large HH: OR 0.14, 95% CI 0.03–0.57, *P* = 0.006, *I* ^2^ = 31%, Large to very large HH: OR 0.21, 95% CI 0.09–0.51, *P* = 0.0005, *I* ^2^ = 0%, Small HH: OR 0.23, 95% CI 0.05–1.01, *P* = 0.050, *I* ^2^ = 38%).

### Postoperative complications

**Fig. 4:**
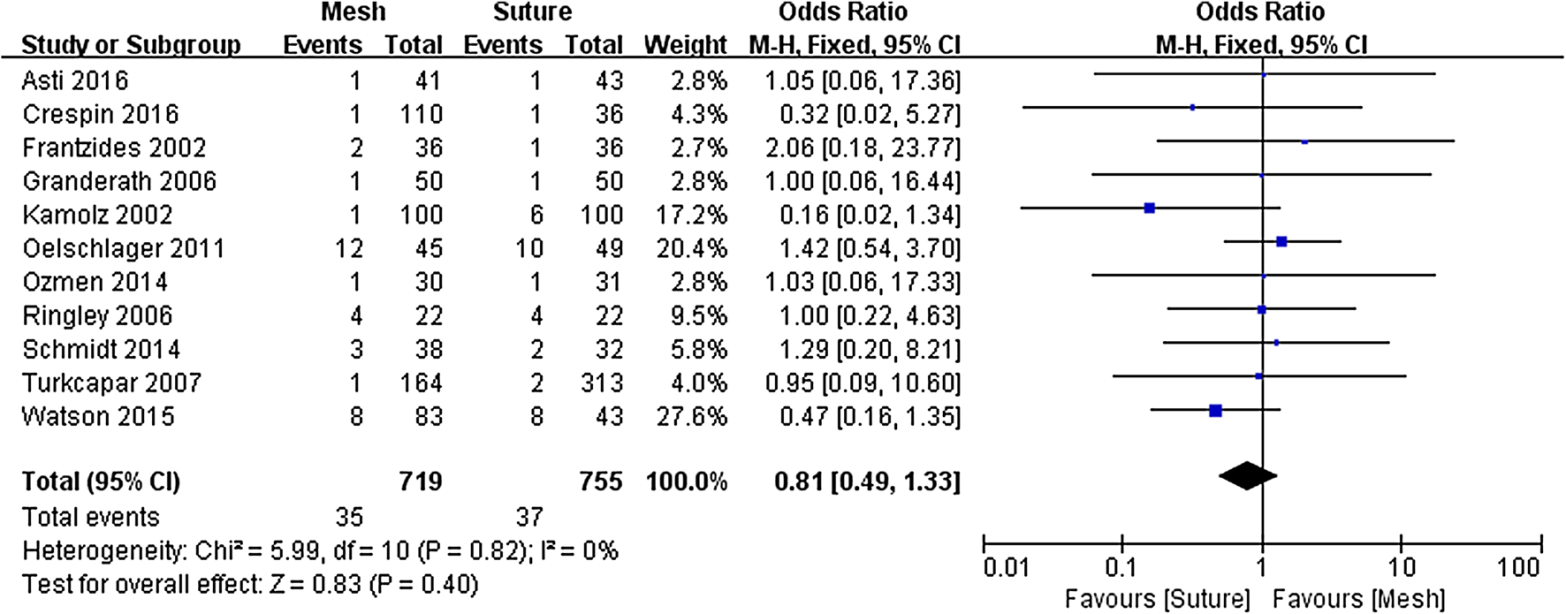
Forrest plot of the odds ratio for postoperative complications

Complication rates of 4.9% were identified in both the mesh-augmented and primary suture repair groups (OR 0.81, 95% CI 0.49–1.33, *P* = 0.400), with no evidence of significant statistical heterogeneity (*χ* ^2^ = 5.99, *P* = 0.740, *I* ^2^ = 0%) in Fig. 4.

**Fig. 5:**
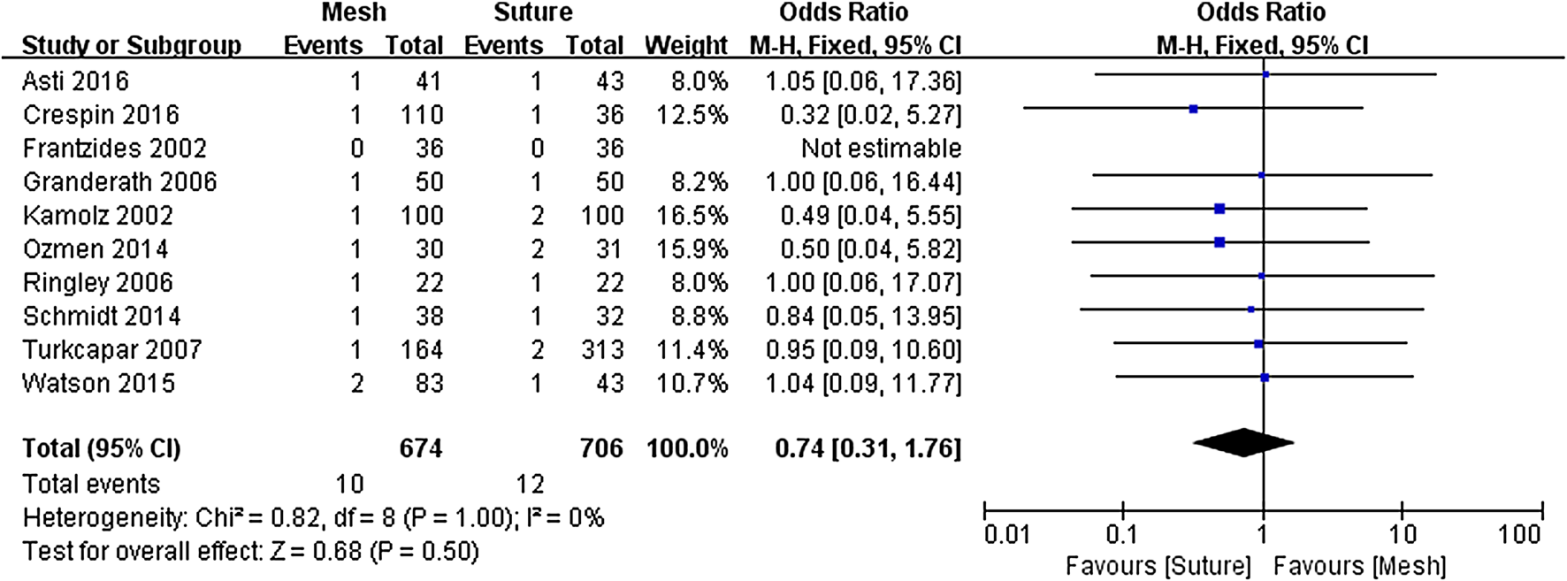
Forrest plot of the odds ratio for postoperative dysphagia

Postoperative dysphagia was analyzed separately to complications for the pooled analysis (Fig. 5). For the RCT reported by Watson et al., the dysphagia rate was calculated using the dysphagia for liquids analog score [17]. No significant differences for postoperative dysphagia were seen for the mesh-augmented group (1.5%) versus primary suture repair (1.7%), (OR 0.74, 95% CI 0.31–1.76, *P* = 0.500), with no evidence of statistical heterogeneity (*χ* ^2^ = 0.82, *P* = 1.000, *I* ^2^ = 0%).

### Mortality

Except for eight cases, no surgery-related death was associated with the two surgical techniques, and the two arms could not be validly compared for mortality. One patient died suddenly 7 days after surgery following a pulmonary embolism or myocardial infarct in Watson et al’s study [17]. One died suddenly from myocardial infarction 14 days after surgery, and two died following massive pulmonary embolism post-discharge in Oeschager’s study [16]. Four died in Asti’s study which is consisted of pneumothorax (*n* = 2), atrial fibrillation (*n* = 1), and acute urinary retention (*n* = 1), without mention of groups [21].

### Quality of life

**Fig. 6:**
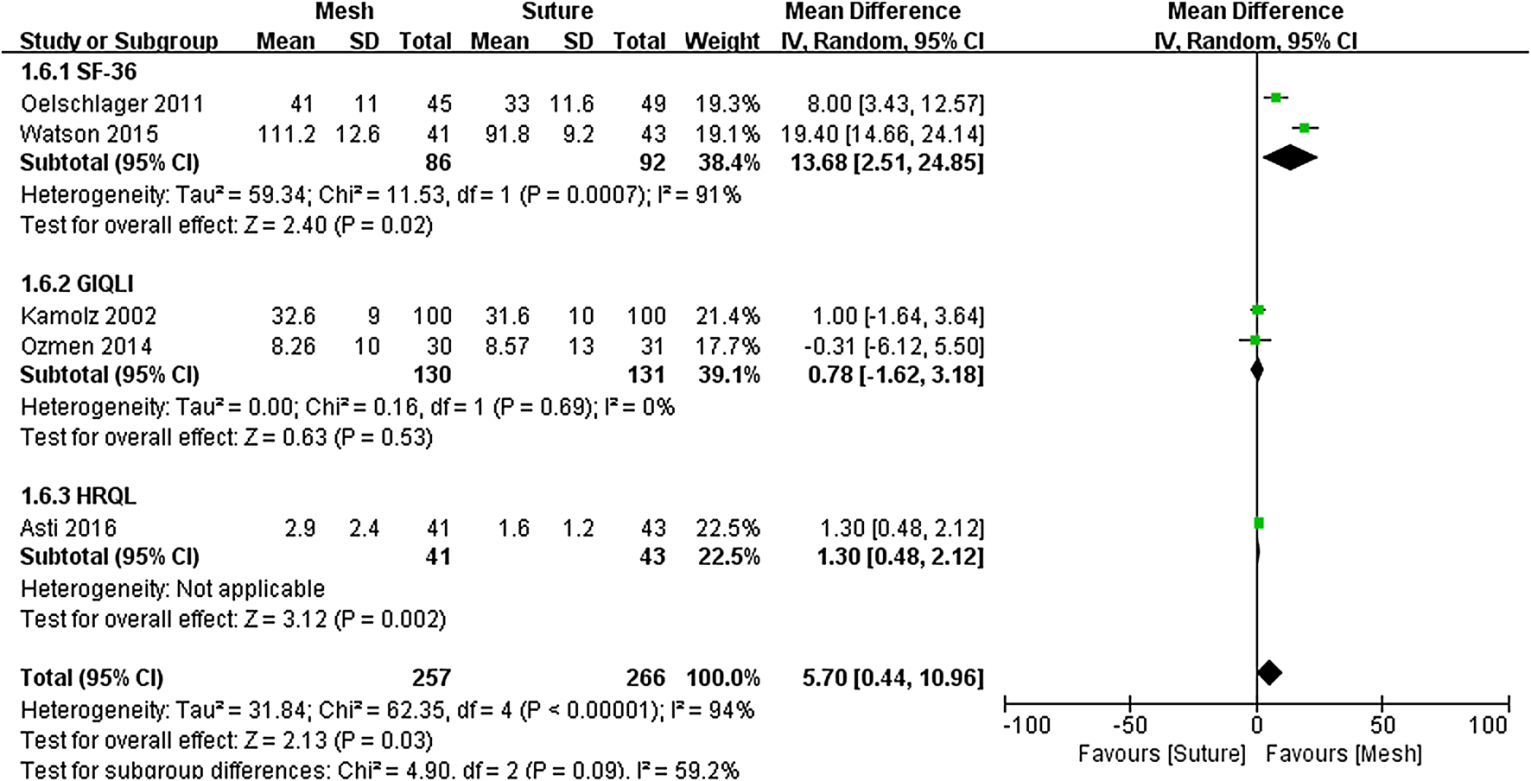
Forrest plot of the mean difference for quality of life

QOL assessment in the included studies general used either the SF-36, GIQLI, or HRQL questionnaires. Two studies used the SF-36 [16, 17], two used GIQLI [19, 22], and one used HRQL [21]. Only permanent mesh implantation studies were available for the GIQLI analysis, and biological mesh implantation studies for SF-36 and HRQL analysis. In this meta-analysis, SF-36 score was calculated by summing up all subscales according to the Oeschager et al’s and Watson et al’s studies. Subgroup analysis (Fig. 6) demonstrated significantly better SF-36 scores following biological mesh-augmentation compared to primary suture repair (MD = 13.68, 95% CI 2.51–24.85, *P* = 0.020), consistently, with a similar result in Asti et al’s study (MD = 1.30, 95% CI 0.48–2.12, *P* = 0.002). However, no significant differences in GIQLI scores were seen for permanent mesh versus suture repair (MD = 0.78, 95% CI −1.62–3.18, *P* = 0.530). There was, however, significant statistical heterogeneity seen in the total QOL assessment (*χ* ^2^ = 62.35, *P* < 0.00001, *I* ^2^ = 94%) and SF-36 analysis (*χ* ^2^ = 11.53, *P* = 0.0007, *I* ^2^ = 91%), but no evidence of significant statistical heterogeneity (*χ* ^2^ = 0.16, *P* = 0.69, *I* ^2^ = 0%) for the GIQLI analysis.

### Improvement of symptoms (analog symptom scores)

**Fig. 7:**
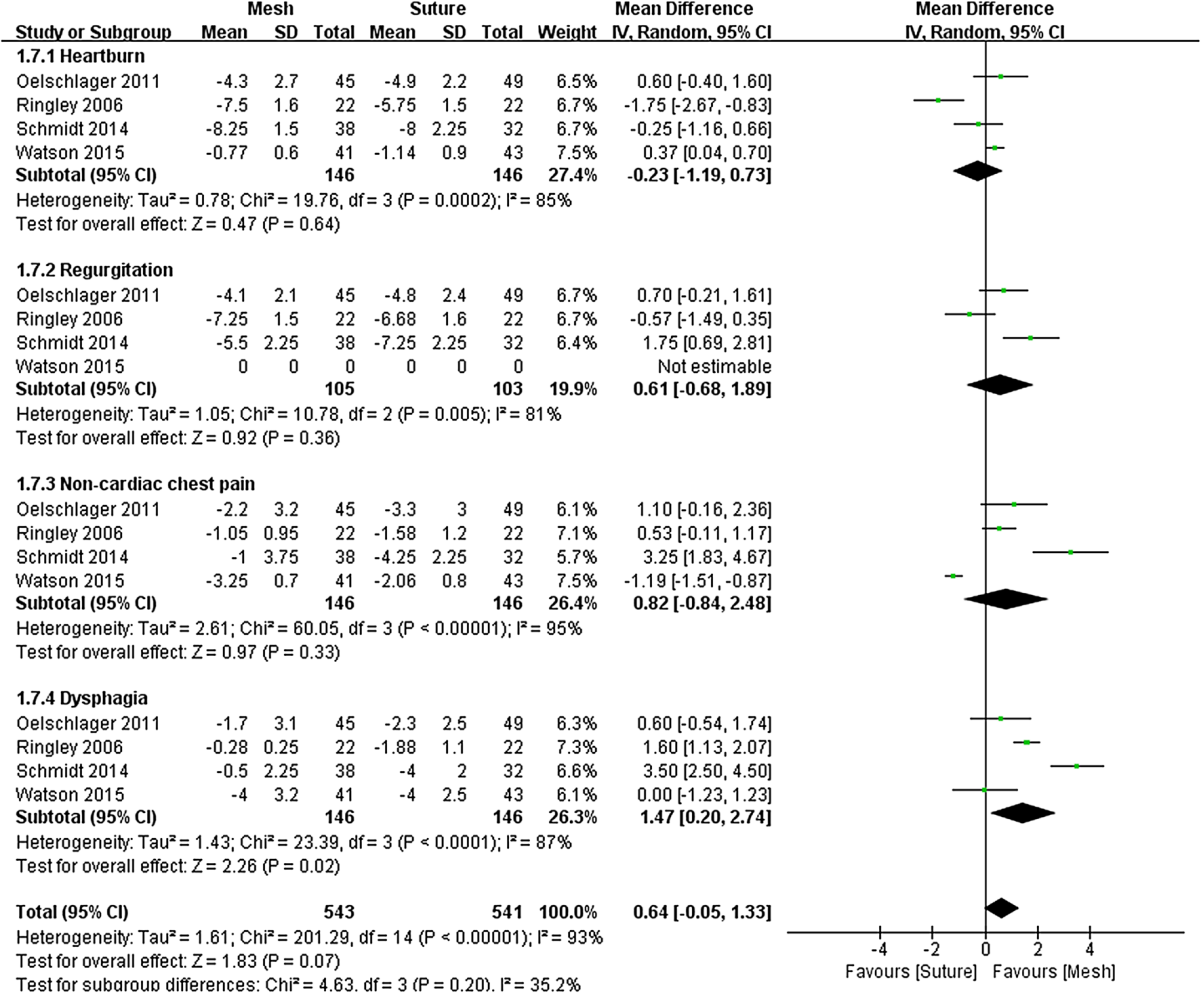
Forrest plot of the mean difference for analog symptoms scores

Four studies [16, 17, 20, 23] used analog scales to report various symptoms before and after surgery, including heartburn, regurgitation, non-cardiac chest pain, and dysphagia. Improvements in the analog symptom scores were calculated and included in a meta-analysis of each symptom (Fig. 7). Meta-analysis revealed no significant difference for heartburn, regurgitation, and non-cardiac chest pain between the two groups. The extent of improvement in dysphagia was greater following repair by sutures alone (MD = 1.47, 95% CI 0.20–2.74, *P* = 0.020). However, excessive heterogeneities were seen for the analysis of all analog symptom scores (*I* ^2^ > 75%).

## Discussion

Laparoscopic fundoplication for GERD commenced in 1991 [27], and has become the “gold standard” surgical approach in patients with medical-refractory GERD [28]. A proportion of patients also present with a very large HH, and this scenario is frequently encountered. HH repair using sutures was initially used for laparoscopic repair. Subsequent studies reported high rates of radiological recurrence of hernias, and this provided impetus to the use of mesh for hiatal repair during repair of large HH, even though most of the identified postoperative hernias were small and asymptomatic. Currently, the use of mesh remains controversial, with little agreement about whether or not to use mesh, what type of mesh (permanent vs. absorbable), mesh configuration, and concern about the risk of mesh-related complications.

Current studies comparing mesh versus suture repair are limited to four RCTs, and a small number of non-randomized case–control studies. Our meta-analysis suggests that the use of mesh is associated with a reduced rate of hernia recurrence compared to primary suture repair at short-term follow-up of 6–12 months. However, longer term data are only available from one RCT [25]. Oelschlager et al. initially reported a significant reduction in hernia rates at short-term follow-up following repair with Surgisis mesh, but no difference at longer term follow-up at 5 years, with recurrence rates of >50% in both groups. The conclusions that can be drawn from Oelschlager et al’s later follow-up report are limited by possible attrition bias, with only 66.7% of patients contributing to late follow-up. Frantzides et al’s RCT [14] showed that PTFE mesh decreased hernia recurrence rates from 22 to 0% for very large HH (*d* ≥ 8 cm) at 2½ years follow-up. While these data suggest that PTFE mesh might reduce hernia recurrence, many surgeons consider PTFE mesh to be associated with an excessive risk of mesh erosion and dysphagia, and have not adopted this approach. Furthermore, later follow-up from this study has not been reported.

Despite the potential for mesh to decrease hernia recurrence rates, arguments continue about the risk and impact of mesh-associated complications [4]. In this meta-analysis, mesh-augmentation did not appear to be associated with an increased risk of complications. However, our analysis only evaluated the overall complication rate, not specific complications, and as follow-up was generally short, late complications such as mesh erosion could not be considered. Mesh-augmentation might lead to increased perihiatal scarring, and thereby contribute to dysphagia. However, at one-year follow-up, the prevalence of postoperative dysphagia was not significantly increased following mesh-augmentation. Even though the impact of different mesh types and mesh configurations was difficult to determine due to the heterogeneity of surgical techniques in the studies reviewed, it is possible that dysphagia is not impacted by mesh repair.

Apart from the integrity of the hernia repair, QOL after surgery is also an important consideration, and global measures of outcome that integrate improvements in symptoms and post-surgical side effects should also be considered when determining surgical success [29]. It is well known that laparoscopic fundoplication for GERD can significantly improve QOL [30, 31]. To compare QOL improvements across the different trial cohorts, we limited the meta-analysis to the SF-36, GIQOL, and HRQL scales. Our results suggested that mesh repair was associated with greater short-term improvements in the SF-36 scales, and the magnitude of the OR was slightly in favor of the mesh group when using HRQL, although differences were not seen for the GIQOL score. The data underpinning the SF-36 and HRQL scores were from studies that used biological mesh, whereas the GIQOL data came from studies that used a permanent mesh. It is feasible that the different types of mesh impact QOL differently, and that the use of a biological mesh reduces or eliminates the risk of problems which might accompany excessive scarring around a permanent mesh material. However, the data in our study are not definitive, and this is an area for future research. Moreover, our analysis suggests that mesh-augmentation does not impact many of the symptoms assessed by analog scales, including typical symptoms of GERD (heartburn and regurgitation) and atypical symptoms like non-cardiac chest pain. However, dysphagia appeared to be impacted by mesh-augmentation, with lower levels of post-surgery improvement, compared to a primary suture repair, although these results should be considered cautiously as the heterogeneity was high (*I* ^2^ = 87%).

Our meta-analysis has some inherent limitations. Seven of the eleven studies included in the meta-analysis were not randomized. This increases the risk of selection and detection bias. A wide variety of surgical techniques were also used, mesh shapes and mesh types were used (biological and permanent mesh), and differences between techniques were beyond the scope of this meta-analysis. In addition, outcomes for most studies were short term, with only one study reporting longer term outcomes, and these outcomes differed significantly from the earlier reported short-term outcomes.

In conclusion, our meta-analysis suggests that mesh-augmentation should at least be considered for repair of HH. Compared to primary suture repair, mesh repair of HH appears to be associated with a lower recurrence risk at short-term follow-up, a similar pattern of complications and reflux symptoms, but perhaps offset by more dysphagia. Biological mesh was also associated with better short-term QOL scores. However, the limitations of short-term follow-up in most studies and heterogeneity of surgical techniques, suggest that data should be interpreted cautiously, and the case for routine use of mesh for HH repair is yet to be proven. Further studies should also consider the impact of different mesh configurations and types.

## Abbreviations

CI: Confidence intervals
GERD: Gastroesophageal reflux disease
GERD-HRQL: GERD related Health Related Quality of Life
GQOLI: Gastrointestinal Quality of Life Index
HH: Hiatus hernia
MD: Mean differences
OR: Odds ratio
RCT: Randomized controlled trial
QOL: Quality of Life
SF-36: Short-form 36
